# ISYS (Integrated SYStem): A Platform for Integrating Heterogeneous Bioinformatic Resources

**DOI:** 10.1002/cfg.150

**Published:** 2002-04

**Authors:** Damian Gessler

**Affiliations:** National Center for Genome Resources 2935 Rodeo Park Drive East Santa Fe NM 87505 USA


**Comparative and Functional Genomics**
*Comp Funct Genom* 2002; **3**: 169–175.
Published online 11 March 2002 in Wiley InterScience (www.interscience.wiley.com). **DOI:** 10.1002/cfg.150

**Conference Review**

### ISYS (Integrated SYStem): a platform for integrating heterogeneous bioinformatic resources†

Damian Gessler*
*National Center for Genome Resources, 2935 Rodeo Park Drive East, Santa Fe, NM 87505, USA*

*Correspondence to: National Center for Genome Resources, 2935 Rodeo Park Drive East, Santa Fe, NM 87505, USA. E-mail: ddg@ncgr.org*

**Keywords: Bioinformatics; Integration; integrated system; loose coupling; selection synchronization; Java**

Received: 28 January 2002
Accepted: 7 February 2002

### Introduction

Modern biological discovery is becoming a domain where one increasingly requires a synthesis of disparate biological data to make significant progress. For example, over the life of an investigation it is not uncommon to need to employ QTL (Quantitative Trait Loci) mapping techniques, access sequenced information of the candidate regions, cross species boundaries with sequence homologies via BLAST (Basic Local Alignment Search Tool), seek to relate interesting annotations via gene ontologies, and follow this up with directed gene expression studies. In fact, the multifaceted approach to biological discovery can be seen by noting that the opposite sequence of events is equally feasible.

To be able to deploy this type of investigation, one requires the computational analysis of data at numerous stages. Indeed, while bioinformatic analysis is clearly not solely *sufficient* for biological discovery (except in rare cases), it is becoming increasingly *necessary* as a component in almost all cases. In response to this, much bioinformatic development to date has been in been in either the development of individual, specialized tools and algorithms, or integration at the data level. This is witnessed by the wide variety of expert tools available and the plethora of different standards and databases for cross-data type comparisons.

Yet despite this, moving between even a small number of programs during a single session at the computer can be clumsy and inefficient, and integration at the data level has numerous inherent limitations, including constraints on flexibility, biological context, cost, and timeliness. Integration – or the lack of it – remains one of the fundamental obstacles to the efficient identification, inclusion, analysis, and synthesis of biological data.

### ISYS™: NCGR's Integrated SYStem

There is no one, global integration solution, but a combination of approaches can build systems that really are more efficient than others. Upon studying the problem, it becomes clear that there has been much work and thought in developing specialized bioinformatic tools and unified approaches to data modeling, but relatively little work on a low cost, minimum buy-in, plug-and-play architecture for the

†This review is adapted from a section of the Proceedings of the International Cotton Genome Initiative (ICGI) 2001 workshop and reflects a presentation recently delivered at the 10th Plant, Animal and Microbe Genomes Conference. The content derived from the report for the ICGI is reproduced by kind permission of the ICGI Steering Committee (Roy Cantrell, Chair).

Copyright © 2002 John Wiley & Sons, Ltd.

integration of applications [2]. ISYS, an acronym for Integrated SYStem, approaches the problem by offering a way to integrate computer applications themselves. This should be seen as complementary to the advances of other approaches. Essentially, it allows one to use mouse-clicks and selection events between applications to emulate the experience of working within a single application. Yet ISYS preserves these applications' separate development trajectories: because ISYS integrates legacy code and data sources with arbitrarily complex data models, it value-adds to existing expert specialized code and to existing data integration approaches. ISYS:

- is designed to be a light-weight platform that does not require a costly, up-front investment. ISYS is written in Java and runs client-side on scientists' individual computers. This makes it attainable to a broad number of researchers and developers – the very people who are often directly involved in designing and writing expert specialized applications;
- requires no formalized standards for integration dependent on a wide-scale, industry buy-in. ISYS encourages *convention* over *standardization* as a mechanism to build grass-roots interoperability amongst components. ISYS is not incompatible with standardization efforts, it simply does not require that such industry-wide standards be set before one begins to integrate components;
- allows different technologies to advance at different rates. Computer programs, sometimes called tools, components, or expert code, 'plug in' to the ISYS bus (see below, next section), not to each other. This means that when one wants to incorporate advances in one field, one can plug in the latest ISYS-compatible code without having to make changes to other components. In fact, the new component can immediately experience integrated behavior (see below, this section) with existing components even if they were developed entirely independently of each other. This design feature means that developers can value-add to their code and conserve their efforts (because they can deliver new functionality in combination with other components in an integrated environment), while scientists can customize their desktops to reflect the most recent changes in the fields of their unique interests;
- preserves existing investments by requiring a minimum of effort to get integrated behavior. ISYS does not require changes to the data models of plugged-in components. This makes ISYS very friendly to integrating legacy code, preserving investments, and leveraging work already done. Integration (that is, the act of making code ISYS-compatible) may or may not require changes to a component's source code, with the level of changes varying with the amount and type of integration sought. In general, ISYS 'sits on top of' existing code, so integration usually requires a dozen to a few hundred lines of code to make data available to other programs. When compared to the thousands of lines of code often necessary to develop new applications or significantly expand their functionality, building an integrated environment from existing tools offers substantial savings in investment, while delivering expert code written by experts in their own fields;
- imposes no intellectual property reach-through on code. When you write code to plug into ISYS, that code is yours, to use, restrict, distribute as you see fit. There is no intellectual property reach-through to your code; you have merely adapted it to work in a particular environment (i.e., ISYS), and you can even maintain your code's run-time independence of ISYS in a non-ISYS environment with a single '*if*' statement. NCGR makes the API (Application Programming Interface) available to developers via registration for the Software Development Kit (**http://www.ncgr.org/isys/developers**), so researchers train their own undergrads, graduate students – even themselves –to plug tools into ISYS;
- encourages the wide distribution of plug-and-play code. The preceding features mean that ISYS-adaptability encourages the broad sharing and exchange of specialized, integrated applications. For example, ISYS allows the integration of gene expression viewers and gene ontology viewers, and the ability to unplug either viewer and replace it with a newer one without changes to the rest of the integrated environment;
- presents the equivalent of complex declarative queries in a simple, visual environment. Consider two databases, one of gene expression results and one of gene ontologies, where, for example, one wants to see all results with gene expression ratios greater than five that are involved in DNA

Copyright © 2002 John Wiley & Sons, Ltd. 

repair. It is often costly to go through a complex data-integration process, to be then followed by the need to construct complex declarative queries. Data integration projects require not only the original integration effort, but also regular maintenance and curation. ISYS inserts a level of uncoupling between the informatic projects that really require data integration and the researcher. This moves maintenance and curation costs back to the original data integration providers. ISYS then addresses integration at the application level. For the developer, the costs associated with implementing ISYS functionality can be significantly less than the alternative data integration approach. One such functionality is called *selection synchronization*, and it can be expanded to not only synchronize selection events across independently written programs, but to also send data-hidden events, data-added events, visibility events, etc. Data-events allow one program to filter the display of another. This filtering ability greatly enhances functionality, since now users can get a functional behavior out of the integrated behavior of two programs even if the developers of neither application ever anticipated the scientist's biological question. So, in the above example, ISYS allows researchers to plug in a gene expression viewer and use all of that tool's complicated and sophisticated features to view their gene expression data, and to do so similarly with a gene ontology viewer accessing a gene ontology database. They can then merely select gene symbols of interest in one program and see associated information (e.g., gene ontologies) highlight in the other. Currently, while ISYS can coordinate access to data sources or algorithms written in virtually any language, this type of special on-the-screen visual synchronization requires programs to be written in Java;

- allows one to move from one program to another without having to anticipate all possible paths. ISYS employs a patent-pending technology called DynamicDiscovery™. When programs are 'plugged into' ISYS, they register with ISYS and tell it the type of data they use. ISYS records and coordinates this amongst programs, so when a researcher simply selects something in a program and right-mouse clicks (Ctrl-click on web pages), ISYS returns all those programs that can currently operate on the data. Because single pieces of data can be tagged with many data types (for example, a sequence can be tagged as a sequence, but also with a taxon and an associated gene symbol), there is a rapid combinatoric increase in how one can pursue discovery with data. One may select a sequence anticipating to do BLAST, but then see that one can also go to Gene Ontology and get all related gene symbols in the same biological process. This frees the scientist from always thinking in terms of a bioinformatic recipe, and opens to him or her a point-and-click way to do exploratory discovery. The important point is not that one could not have gone first to Gene Ontology if one had so thought, but that discovery as an endeavor is more fruitful in a richer environment, and this is the environment ISYS attempts to provide. As one combines event synchronization and DynamicDiscovery in the same environment, ISYS begins to bring the same type of point-and-click efficiency one is used to within an application, to between applications; thus the original goal of bringing an application-integration solution to bioinformatics;
- respects legacy data models. Applications present to the ISYS bus the data they choose, and respond to ISYS requests as they choose. This makes ISYS broadly forgiving in the type of applications that can be plugged into the bus, but it also means that ISYS functionality is not imposed on applications: for example, many applications may not be able to do full selection synchronization, so the specific behaviors will vary from application to application in accordance with the developers' design;
- allows integration with web pages. ISYS allows developers to treat web pages as 'components' plugged into ISYS. Users can do Dynamic Discovery in a client-side program and send the data straight to web services. Alternatively, web pages can be 'marked up' with data tags, so users can do DynamicDiscovery straight from web pages and bring the data either back client-side or pass it onto other web pages. Data can be disparate and in different locations –either visually on the web page or even spatially at different web sites –yet with a single mouse-click users can get a list of all those programs currently suitable for those types of data and launch them with a single click. Just like cut-and-paste relieved the need for tiresome and error-prone data-reentry, Dynamic-Discovery on web pages can relieve repetitive

Copyright © 2002 John Wiley & Sons, Ltd. 

cutting-and-pasting. 'Marking up' requires a developer to 'wrap' a web page, but users can also do DynamicDiscovery on single selections of generic text on virtually any web site (currently, ISYS does not support cookies, though the level of functional compromise varies.) As just two examples, a researcher may be reading an article's abstract on a web site, she can then simply select a gene symbol in the text, send if off for a look-up in Gene Ontology and concurrently send it to GenBank to get sequence information. Or she could grab sequence text off a web page and concurrently send it to NCBI for BLASTing and GenScan for gene prediction. The process of 'marking up' allows developers to add what are essentially virtual hyperlinks to a web page. The Java code to do this is relatively simple and resides client-side, so with a little programming individuals can customize web page behavior to their needs. It used to be that users only had those hyperlinks presented to them by the web page author, but now non-authors can mark up web pages so they decide where new links should be: importantly, these 'links' are content-sensitive, so instead of just being a static hyperlink, they link you to whatever services are currently registered with ISYS.

### Software bus and the loose-coupling architecture

ISYS itself is invisible to the user. ISYS can be thought of as a *bus*, that is, a piece of middleware that acts like an orchestra conductor, coordinating events and movement between applications. When one adapts one's program to plug into ISYS, one merely adds some code that allows the program to register with ISYS and act appropriately when a user does something like selecting data, hiding data, etc. ISYS does not require one to implement all these features, since some data models may be less amenable to certain operations that others. When a user in *another* application does something relevant to your application, ISYS sends your application an event and gives it a chance to take some action. Because your application has registered on the bus as being relevant for certain data types, whenever a user does DynamicDiscovery on that data type your application is listed as a viable route for invocation. Because event synchronization between components needs to be meaningful, ISYS establishes synchronization between components only upon DynamicDiscovery. Applications can always desynchronize if they choose. Because applications do not synchronize directly with each other (nor do they directly handle DynamicDiscovery themselves), they need not be concerned with what else is plugged into the bus. This type of architecture is called 'loose coupling' [1] and it is what gives developers and scientists the flexibility to plug-and-play different components in a simple coordinated fashion. ISYS has the notion of *static services* whereby a component can demand that another specific application be present. In this manner, if a component requires tighter integration it can demand it, but it is not mandated by ISYS.

### Modules can be upgraded without rewriting software

The loose coupling architecture achieves integration by having components interact via the common ISYS API, not directly with each other. Thus, for example, if an application receives an *ItemSelected* event (altering it that it should now highlight matching items for the user), it does not care if that event was generated by a gene expression viewer, a database browser, a generic viewer, or whatever application. Components are essentially ignorant about why they are requested to do something. Because of this, if the user unplugs the gene expression viewer and replaces it with a newer one – or even replaces it with an entirely different application like a thesaurus/glossary program – and this program also generates an *ItemSelected* event that is sent to the receiving application, then the application processes the event in the same way; that is, there are no code changes necessary to get instant visual integration with this new application.

### DynamicDiscovery

DynamicDiscovery is the process whereby users can invoke new programs based on the type of data they are currently selecting. Upon invocation, event synchronization is established (Figure 1). DynamicDiscovery allows one program to send data to another program, and minimizes the chance that the data will be inappropriate. ISYS runs all

Copyright © 2002 John Wiley & Sons, Ltd. 

visually synchronized programs within the same process space inside a single Java Virtual Machine, though it can easily interact with non-synchronized programs in other process spaces. This means that developers can take advantage of Java interfaces to display views of their data model to other programs without the actual transfer of data. This means that formats such as XML, or technologies such as RMI (Remote Method Invocation) or CORBA (Common Object Request Broker Architecture) are not necessary. Often, 'data transfer' is achieved simply by passing memory references, without the data itself ever being copied. This can allow extremely efficient inter-application communication. A disadvantage is that memory sharing and CPU allocation within a process space does not benefit from the many operating system level inter-process security and robustness measures, so errant or malicious applications can destabilize ISYS or corrupt data. The latter problem can be minimized by a strict

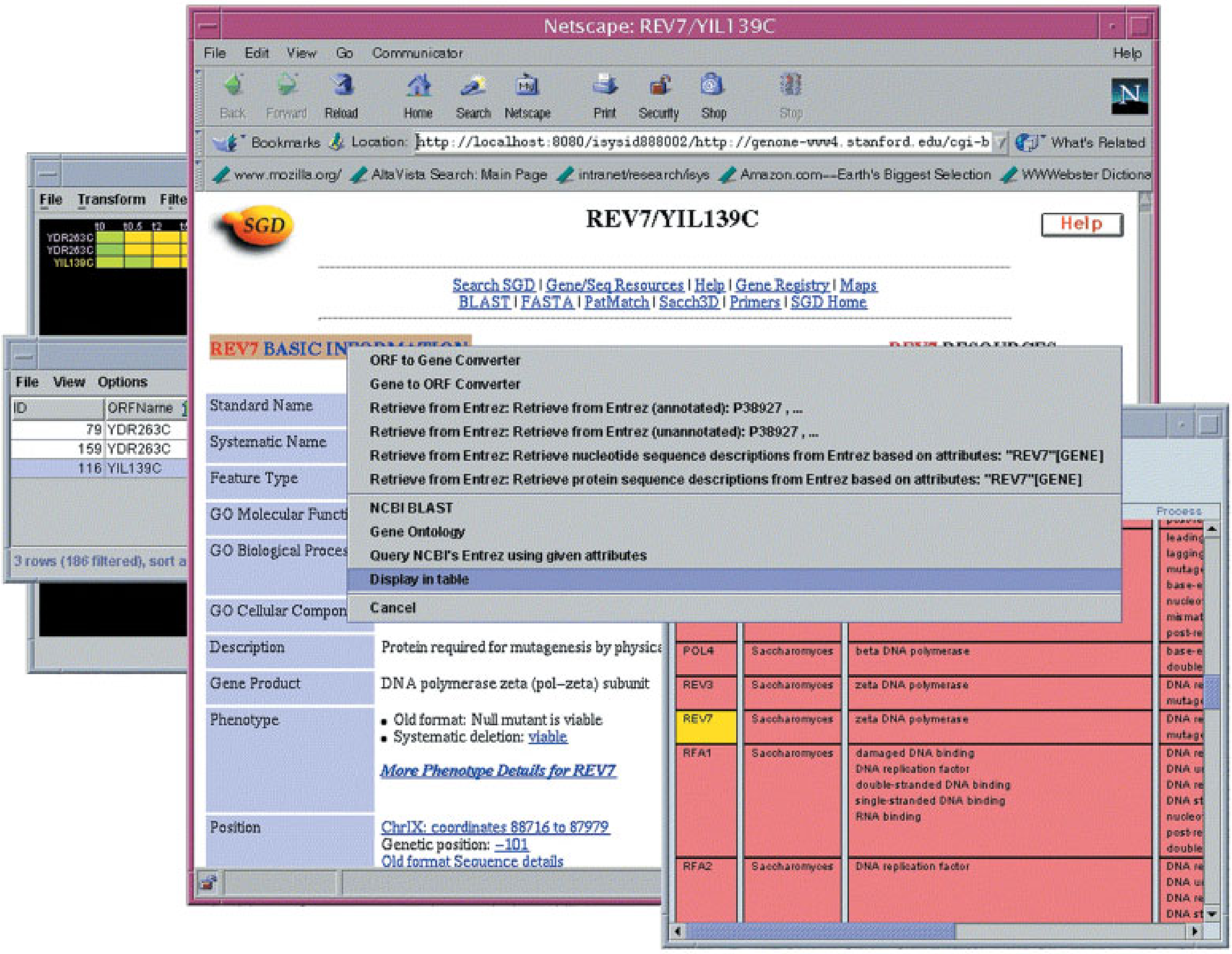

**Figure 1.** Research using four different, integrated tools. The researcher has gene expression results with simply ORFs (open reading frames) as identifiers (background tool). Via ISYS, the information is sent to a database table view, which maps ORFs to gene symbols, and then to the Berkeley Drosophila Genome Project's gene ontology viewer (red table in foreground). All three tools are synchronized, highlighting the gene symbol REV7 (YIL139C). The researcher has then filtered back to the original set so it only includes those genes included in DNA repair. The researcher is also viewing information about REV7 on the *Saccharomyces* Genome Database's web page and has invoked DynamicDiscovery (gray menu) directly on the web page. The researcher can now extract information and send to the list of registered ISYS services. Reproduced by permission of the International Cotton Genome Initiative (ICGI) Steering Committee

Copyright © 2002 John Wiley & Sons, Ltd.

adherence to good object-oriented programming standards.

### Data types and attributes

At the heart of synchronization and Dynamic-Discovery is the tagging of data as different data-types. This is done by employing Java interfaces to tag classes with *attributes*. Just like a cup on your desk may be white, made of porcelain, exist in some space-time framework, and belong to you 'all at the same time' – i.e., without any explicit data structure – ISYS allows that tagging of data with disparate biological meanings by similar 'all at the same time' attributes. This independence of attributes means that you can unplug gene expression programs without if affecting the functionality of your gene ontology browser on an given piece of data, and vice versa. Developers can add attributes in a local manner as they see fit, so ISYS does not dictate any global biological data model.

Within a top-level independence of attributes, other attributes can be hierarchy nested, and all attributes can mandate the implementation of arbitrarily complicated (or simple) *methods* or functions to ensure that they are used and interpreted correctly. For example, a developer may add the new attribute *MyComplicatedMethylatedSequence* under the existing attribute *SequenceText*. Implementations of that new attribute on data will have to satisfy methods dictated by *SequenceText* (*e.g*., a method called *getSequenceText*() which returns the sequence as a text string), while at the same time specifying any new methods. Legacy programs that were registered on the bus as listening for *SequenceText* will continue to work, while new programs that register to listen for *MyComplicatedMethylatedSequence* will also interact with programs recognizing just *SequenceText* (since *MyComplicatedMethylatedSequence* is a type of *SequenceText*), while also offering new functionality for their new type of data. Synchronization is accomplished on the *values* of the attributes, as returned by the attributes' methods.

### An example of ISYS in comparative genomics

NCGR and international centers of the Consultative Group on International Agricultural Research (CGIAR) have recently completed a pilot project to connect the CGIAR's International Crop Information System (ICIS) to ISYS with the inclusion of a new Comparative Map Viewer. ICIS stores hundreds of megabytes of crop genealogical and associated data. The Centers of the CGIAR want this data available to a variety of analysis tools, thereby leveraging the value of their data by being able to analyze it in an integrated, customizable manner. The Comparative Map Viewer allows the visualization of syntenous regions between linkage groups either within or between species. Via ISYS services, the tool can access data directly from ICIS and also from more generic sources. Both the Comparative Map Viewer and ICIS plug into ISYS, so, for example, CGIAR researchers can view their mapping data and simply click on annotated regions to search amongst Gene Ontologies and GenBank, or even click on unannotated, physical regions and send sequences to gene prediction algorithms like GenScan.

### The larger picture

There is a common theme in how bioinformatics operates in research communities. Efficient data utilization tends to spring from 1. community-wide resources, such as server/web configurations, and 2. client-side analysis tools that reflect each scientists' needs. CottonDB, SGD (Saccharomyces Genome Database), Flybase, MGI (Mouse Genome Informatics), TAIR (The Arabidopsis Information Resource, a collaboration between Stanford University's Carnegie Institute of Washington and NCGR), and numerous others provide important resources to their research communities. These resources gain even greater functionality when 1. and 2. are combined, presenting an integrated client-server package. ISYS is designed so that these different technologies can proceed at different rates, with the input and contribution of different people, while leaving the basic power of integration configuration in the hands of the scientists and developers themselves.

NCGR is currently integrating their biochemical network database and software tool called PathDB into ISYS. Curators enter biochemical pathway data into PathDB's database as atomic reaction steps. Currently, information from over 900 papers on Arabidopsis have been entered into PathDB, and while the total number of 'pathways' is still

Copyright © 2002 John Wiley & Sons, Ltd.

small (about 162), the Arabidopsis information complements entries spanning 202 taxa and thousands of proteins, compounds, metabolites, and literature citations. When completed, a researcher will be able to query the database by numerous means, and the program will then rebuild and display networks on-the-fly. Because reactions, and not pathways, are the basic building blocks, the computer can identify new network connections (essentially, hypotheses at this stage) not known by previous researchers. Once integrated into ISYS, users will be able to go from gene expression displays to biochemical networks simply by selecting and clicking on their results. For availability of ISYS, please visit **http://www.ncgr.org/isys**.

### Acknowledgement

ISYS reflects the work of many people. Of special note and recognition for their pioneering roles are Adam Siepel, Andrew Farmer and Andrew Tolopko.

### References

1. Siepel AC, Farmer AD, Tolopko AN, *et al.* 2001. An integration platform for heterogeneous bioinformatic software components. *IBM Systems Journal* **40**: 570–591.
2. Siepel A, Farmer ATolopko A, *et al.* 2001. ISYS: a decentralized, component-based approach to the integration of heterogeneous bioinformatic resources. *Bioinformatics* **17**: 83–94.

Copyright © 2002 John Wiley & Sons, Ltd.

## References

[PDF_00526] Siepel A., Farmer A., Tolopko A., Zhuang M., Mendes P., Beavis W., Sobral B. (2001). ISYS: a decentralized, component-based approach to the integration of heterogeneous bioinformatics resources.. Bioinformatics.

